# Editorial Notes

**Published:** 1940

**Authors:** 


### Editorial Notes

**Nuffield Provincial Hospitals Trust.**

A CONFERENCE was held in Bristol, on 11th October, which may well prove to have far-reaching results for the hospitals in this area in the future. It was called by the Lord Mayor of Bristol, to meet four representatives of the above Trust, Mr. W. M. Goodenough the Chairman, Alderman W. Hyde the Secretary, Sir Farquhar Buzzard and Dr. A. Q. Wells, the Chairman and Secretary of the Medical Advisory Council. The meeting was held at the Council House and was attended by about eighty persons, representatives of all the medical charities and institutions in the district. Members of the lay committees and medical staffs of the larger hospitals had an opportunity for a further and smaller conference earlier in the day.

An explanation of the Trust and its functions may be necessary. A commission appointed in 1937, by the British Hospitals Association, with Lord Sankey as Chairman, made the following recommendations :—

1. The division of the country into Hospital Regions.
2. The formation in each region of a Voluntary Hospitals Regional Council to correlate hospital work.
3. The formation of a Voluntary Hospitals Central Council to co-ordinate the work of the Regional Councils.

Not much progress, however, was made before the war started, and there was no fund, such as the King Edward's Hospital Fund in the London area, to cater for the needs of the provincial hospitals. The war threatened the very foundations of the voluntary hospitals. At this juncture, at the end of 1939, Lord Nuffield, with his well-known generosity and eye for a great and good cause, announced his willingness to give share units in Morris Motors Ltd. to the value of a million pounds to encourage the development of the regional councils and the hospitals within their sphere of responsibility. A beginning was made in the Oxford district, and the deputation, after careful preparation in which the Lord Mayor (Alderman Burgess) took a great interest, was visiting Bristol to encourage the formation of a regional council here.

After some excellent remarks by the Lord Mayor in which he prayed all present to be willing to think and care for the provision of hospital services as a whole, even if that might not mean the aggrandizement of the particular institution in which they took a personal interest, Mr. Goodenough and Sir Farquhar Buzzard explained the scheme, and invited questions. It was made clear that the Ministry of Health were in favour of regional councils: what was aimed at was co-operation instead of competition. Municipal and voluntary hospitals should work together as a team. If a beginning were made in Bristol, it would be entirely autonomous; no one had a cut-and-dried plan to foist upon them, and how many of the surrounding counties and neighbouring towns joined in depended on how attractive the scheme could be made. Hospitals with special methods of treatment were not to be asked to abandon these.

The meeting unanimously agreed to accept the proposals, and it was entrusted to the Lord Mayor, the Chairman of the Health Committee (Alderman Milton), and the Chairman of the Board of the Bristol Royal Hospital (Col. P. G. Robinson), to draw up a list of those who should be asked to sit upon the Bristol Regional Council. After votes of thanks to the deputation and to the Lord Mayor, the meeting terminated, getting through its business with no waste of time and a remarkable degree of concord. The new Regional Council will take the place of the Bristol Hospitals Council, which has been in a state of suspended animation for the past year or two.

**Save the Children Fund.**

THE Save the Children Fund, founded in 1919, exists to prevent child suffering and to promote child welfare, irrespective of the race, nationality or creed of the children helped. Its war-time work includes the establishment of residential and non-residential nursery centres in collaboration with the Ministry of Health and the Board of Education, and the emergency care of children who are victims of air raids. Its sister organization in the United States, the Save the Children Federation, of New York, announces that an American Medical Association wishes to send funds to assist, through the Fund, the children of British medical men and women who are suffering on account of the war. For particulars apply to Dr. J. A. Nixon, 7 Lansdown Place, Clifton, Bristol 8.

